# A new gene-based association test for genome-wide association studies

**DOI:** 10.1186/1753-6561-3-s7-s130

**Published:** 2009-12-15

**Authors:** Alfonso Buil, Angel Martinez-Perez, Alexandre Perera-Lluna, Leonor Rib, Pere Caminal, Jose Manuel Soria

**Affiliations:** 1 Unitat de Genomica de Malalties Complexes, Institut de Recerca de l'Hospital de la Santa Creu i Sant Pau, Barcelona, 08025, Spain; 2 Centre de Recerca en Enginyeria Biomedica, Departament d'Enginyeria Sistemes i Automatica Industrial, Universitat Politecnica de Catalunya, Pau Gargallo, 5, 08028 Barcelona, Spain

## Abstract

Genome-wide association studies are widely used today to discover genetic factors that modify the risk of complex diseases. Usually, these methods work in a SNP-by-SNP fashion. We present a gene-based test that can be applied in the context of genome-wide association studies. We compare both strategies, SNP-based and gene-based, in a sample of cases and controls for rheumatoid arthritis.

We obtained different results using each strategy. The SNP-based test found the *PTPN22 *gene while the gene-based test found the *PHF19*-*TRAF1*-*C5 *region. That suggests that no single strategy performs better than another in all cases and that a certain underlying genetic architecture can be delineated more easily with one strategy rather than with another.

## Introduction

The last three years have witnessed an enormous increase in genome-wide association studies (GWAS). The original idea behind these studies is to genotype some hundreds of thousands of single-nucleotide polymorphisms (SNP) in a sample of cases and controls for a given disease, and then look for association between every SNP and the disease. Thus, the researcher has to perform as many statistical tests as SNPs that he has. In other words, the unit of association is the SNP. In this paper, we propose a statistical test for GWAS in which the basic unit of association is the gene--that is, the researcher performs one statistical test per gene. The idea is to combine the genetic information given by all the SNPs in a gene to obtain a more informative result.

Our test presents two advantages compared with the classical SNP-based test. On the one hand, it suffers much less from the problem of multiple testing because we are doing around 20,000 tests instead of half a million. On the other hand, we expect that in those genes with multiple functional variants, the gene-based test will be more powerful than the SNP-based test [1]. However, the gene-based test presents some drawbacks. Because genes do not cover the whole genome, the gene-based test does not use all of the SNPs available. Moreover, the gene-based test is not as simple as the SNP-based test and therefore it requires more computational resources. Our goal was to compare the results using a SNP-based test to those obtained using a new gene-based test for GWAS.

To perform the comparison, we used data from individuals with and without rheumatoid arthritis (RA)--a systemic autoimmune disease characterized by inflammation of the synovial tissue and local articular damage. Studies of RA heritability in two European populations reported that 60% of the disease variance can be attributed to genetic factors [2]. Linkage and association studies have demonstrated that alleles at the human leukocyte antigen (HLA) class II gene *DRB1 *have a strong effect on the risk of RA [3]. However, these variants do not explain all of the heritability. It is possible that loci not linked to the HLA region play an important role in RA susceptibility. Several studies have reported genes or genomic regions related to RA susceptibility outside the HLA region [4-8].

## Methods

### The data

We used the subset of the data from the North American Rheumatoid Arthritis Consortium (NARAC) study provided for Genetic Analysis Workshop 16 [9]. It consisted of 868 cases and 1194 controls. The Ilumina 550 k chip (545,080 SNPs) was genotyped in the whole sample. Before the analysis, we cleaned the data. For example, we excluded SNPs with a call rate smaller than 0.95 (18,627 SNPs), minor allele frequency smaller than 0.01 (23,047 SNPs), or with a *p*-value for the Hardy-Weingberg equilibrium test smaller than 1 × 10^-5 ^(1,342 SNPs). We also excluded six individuals with sex genotype inconsistencies. All of the individuals had a call rate greater than 0.95. All of our analyses were done with 'affection status' as the trait of interest and 'sex' as a covariate.

### SNP-based test

We estimated the association between the trait RA and a SNP using logistic regression assuming a codominant model for the genetic effect of every SNP. We used the *p*-value of the test as a measure of statistical significance. We used the GenABEL software package [10].

### Gene-based test

To perform a gene-based test, the first problem is to define the genes and to assign SNPs to the genes. Also, it is important to be sure that the physical positions of the genes and SNPs refer to the same annotation release. We used the NCBI build 129 release 36.3 for both genes and SNPs. We accepted that a SNP was in a gene if it was inside the gene plus or minus 5,000 base pairs. We analyzed 21,672 genes that contained 272,604 SNPs. That means that around half of the available SNPs were not assigned to genes and, thus, they were left out of the gene-based analysis.

The paradigm for the proposed gene-based test has three steps [11]:

1. Estimate the genetic similarity among individuals based on the genotypes of the SNPs in a given gene.

2. Cluster the individuals in groups by genetic similarity.

3. Test the association between the groups of individuals and the trait of interest.

In the first step, we used the Gower distance. Also known as Gower's coefficient [12], it is a measure of the similarity between two individuals based on the information given by a set of quantitative or qualitative variables. We realized that, in the special case of SNP genotypes, Gower distance is the same as the identity-by-state (IBS) multilocus measure. IBS allele sharing is a measure of genetic similarity between two individuals. Given the genotypes of two individuals at a given SNP, the IBS between them is 0, 1, or 2 depending on whether they share 0, 1, or 2 alleles at that SNP. This measure can be extended to several SNPs by adding the IBS for each locus and dividing by twice the number of loci [13]:

where L is the number of loci considered in the calculation; *g*^*l*^_*i *_and *g*^*l*^_*j *_are the genotypes of individuals *i *and *j*, respectively, at the *l*^th ^locus (*l *= 1, ..., L); and IBS^*l*^_*ij *_is the IBS between *i *and *j *at locus *l*. We estimated this similarity measure for every pair of individuals at every gene. Thus, the result of the first step is a distance matrix among individuals in a given gene.

In the second step, the distance matrix is used for finding groups of individuals with similar genotypic distribution in the given gene. This clustering is performed in a hierarchical procedure by means of a complete linkage agglomerative algorithm. Complete linkage evaluates distances between two groups as the distance of their most distant pair of individuals. We divided the individuals in three groups of similarity. To test the effect of the chosen cluster algorithm on the results, we repeated the analysis in chromosomes 1, 6, and 9 using two other cluster algorithms: hierarchical average linkage agglomerative algorithm and spectral clustering. Average linkage evaluates distances between two groups as the mean distance between individuals of each cluster.

Spectral clustering is a method that defines *k *clusters on a set of *n *data points representing arbitrary objects. It is based on the spectral decomposition of the normalized Laplacian graph defined from a similarity matrix among the objects [14]. In our case, the objects are the individuals and the similarity matrix is the genetic similarity matrix defined above.

Finally, in the third step, association between the groups of individuals and the phenotype of interest was estimated using logistic regression with the group as a factor.

## Results

### SNP-based association

With 545,080 SNPs, the strict Bonferroni *p*-value for a genome-wide significance of 0.05 for the SNP-based test is *p *= 9.17 × 10^-8^. However, because there is some amount of linkage disequilibrium among them, the effective number of tests is smaller. A recent study has estimated that the number of effective independent tests done with the Illumina 550 k chip is 324,559 [15]. That means that the adjusted critical *p*-value is 1.5 × 10^-7^. As expected, there are many SNPs showing statistically significant association with RA in chromosome 6. Specifically, there are 213 statistically significant SNPs, that cover 74 genes, all of them in the HLA region, in 6p21. In addition, the SNP rs2476601 in chromosome 1 has a *p*-value of 2.04 × 10^-8^. This SNP lies on the *PTPN22 *gene, which has been previously associated with RA [4].

### Gene-based association

With 21,672 genes analyzed, the strict Bonferroni *p*-value for a genome-wide significance of 0.05 for the gene-based test is *p *= 2.3 × 10^-6^. The gene-based association test presents 60 statistically significant hits, with *p*-values as low as 1 × 10^-74^, in 6p21, as expected. The number of SNPs in these genes ranges from 2 to 37, with an average of 7.7. Table 1 shows the top gene-based association results outside of chromosome 6.

**Table 1:** Gene-based association results out of chromosome 6

Symbol	Map location	No. SNPs analyzed on gene	*p*-Value of the gene-based test for the gene	Minimum *p*-value obtained for any SNP on the gene using the SNP-based test
*PTPN22*	1p13	6	0,11	2.04 × 10^-8^
*PHF19*	9q33.2	4	9.61 × 10^-7^	7.07 × 10^-6^
*FLJ11151*	16p13.12	40	3.98 × 10^-6^	2.69 × 10^-4^
*GLT8D3*	12q12	6	8.37 × 10^-6^	4.92 × 10^-2^
*LSM3*	3p25.1	5	1.24 × 10^-5^	2.31 × 10^-4^

The *PHF19 *gene lies in 9q33.2--an area that has been associated recently with RA [5,8], and shows a clear statistical significance with the gene-based test, but not with the SNP-based test. Three other areas that show suggestive association with RA are 16p13.12, 12q12, and 3p25. These areas contain previously described quantitative-trait loci for RA, which can be found at the UCSC genome browser database [16].

To test the sensitivity of the new method to the cluster algorithm, we repeated the analysis of chromosomes 1, 6, and 9 using two other cluster algorithms: hierarchical agglomerative average algorithm and spectral clustering. In the HLA region, the average clustering found 42 of the 60 genes found by the original cluster method and 12 genes not detected by the original. On the other hand, the spectral clustering found 54 of the 60 genes found also by the original cluster method and 28 not detected by the original. Outside of the HLA region, the gene *PHF19 *on chromosome 9 was found by the spectral, but not by the hierarchical average algorithm. None of the cluster algorithms found the *PTPN22 *gene that was found by the SNP-based test.

A possible question associated with the new method is whether it is sensitive to the number of SNPs in the gene. We discretized the number of SNPs in quartiles and tested the association between this variable and the -log_10 _of the *p*-value by means of a one-way ANOVA. We did not find an association between the two variables (*p *= 0.14). We repeated the analysis for each chromosome separately and none of them gave a statistically significant association.

## Discussion

We have compared the results of a new gene-based test with the results using a standard SNP-based test in a sample of cases and controls for RA in a GWAS. The procedure of the test was to group the individuals by genetic similarity in a given gene and test whether the distribution of groups is different between the cases and the controls. In some ways, this approach is related to the association with haplotypes, because both approaches combine genetic information from several SNPs. However, haplotype-based methods suffer from a statistical problem--that is, the abundance of rare haplotypes. To solve this problem, different strategies that reduce the number of haplotypes have been proposed, i.e., haplotype clustering [17] or inference of ancestral haplotypes [18]. Our approach shares this idea, but is much simpler because it works directly with genotypes instead of haplotypes. In fact, in a previous study, we used this approach with data of a complete resequenced gene and we were able to group the individuals in the same way that when using the ancestral haplotypes [11].

As expected, both gene-based and SNP-based tests gave many statistically significant results in the HLA region. However, both tests gave different results outside of the HLA region. The SNP-based test found the *PTPN22 *gene while the gene-based test found the *PHF19 *gene. A possible explanation is that different underlying genetic architectures can be more easily detected with one strategy but not with the other.

The gene-based test has some limitations. One of these limitations is that, as defined here, it uses only half of the genetic information available. That is a consequence of how we assign SNPs to a gene: a SNPs belongs to a gene if it is inside the gene plus or minus 5,000 base pairs. Some researchers solve this problem assigning every SNP to the nearest gene, no matter how far it is from the gene. We think that a better strategy is to change the gene-based test to a region-based test. In our test, we used the gene as the unit of the analysis, but a genomic region of a fixed size could be used instead. Then, the test could be applied with a 'sliding window' strategy and utilize all the available genetic information.

Other limitations are related to the clustering algorithm. On the one hand, different algorithms can give slightly different results and, on the other hand, the test does not take into account the uncertainty of the clustering process. To measure and, eventually, correct this bias, we are planning to evaluate the effects of using different cluster algorithms and to use re-sampling techniques, as bootstrap or permutations, to estimate the statistical evidence of the test.

## Conclusion

We performed a GWAS for RA affected status first with a standard SNP-based association test and second with a new gene-based association test. Both strategies gave a large amount of statistical evidence of association in 6p21 and little evidence outside of this region. With the SNP-based test we found a SNP in the *PTPN22 *gene that was not found with the gene-based approach. On the other hand, with the gene-based test we found an area in 9q33.2 that was not detected by the SNP-based test. We do not think that one test is better than the other. They simply use the genetic information in a different way. We consider both tests as complementary.

## List of abbreviations used

GWAS: Genome-wide Association Studies; HLA: Human leukocyte antigen; IBS: Identity by state; RA: Rheumatoid arthritis; SNP: Single-nucleotide polymorphism.

## Competing interests

The authors declare that they have no competing interests.

## Authors' contributions

AB designed the study, participated in the statistical analysis, and drafted the manuscript. AM-P carried out most of the statistical analyses. AP-L carried out the spectral clustering analysis and participated in the writing of the manuscript. LR participated in the statistical analysis. PC and JMS participated in the design of the study and reviewed the manuscript.
